# Ectopic insulinoma diagnosed by ^68^Ga-Exendin-4 PET/CT: A case report and review of literature

**DOI:** 10.1097/MD.0000000000025076

**Published:** 2021-04-02

**Authors:** Xiaona Zhang, Hongwei Jia, Fengao Li, Chunyun Fang, Jinyang Zhen, Qing He, Ming Liu

**Affiliations:** a Department of Endocrinology and Metabolism, Tianjin Medical University General Hospital; b Nankai University State Key Laboratory of Medicinal Chemical Biology, Nankai University, Tianjin, China.

**Keywords:** ^68^Ga-DOTATATE PET/CT, ^68^Ga-Exendin-4 PET/CT, case report, ectopic insulinoma, hypoglycemia

## Abstract

**Rationale:**

Ectopic insulinomas are extremely rare and challenging to diagnose for clinicians. Precise preoperative localization is essential to successful treatment.

**Patient concerns:**

A 23-year-old man presented with a 1-year history of recurrent hypoglycemia.

**Diagnosis:**

Examinations in the local hospital did not reveal any pancreatic lesion. After admission, a fasting test and a 5-hour oral glucose tolerance test (OGTT) suggested a diagnosis of endogenous hyperinsulinemic hypoglycemia. Enhanced volume perfusion computed tomography (VPCT) revealed 2 nodules in the tail of the pancreas, a nodule in the gastric antrum, and a nodule in the hilum of the spleen. To differentiate which nodule was responsible for hypoglycemia, we performed ^68^Ga-Exendin-4 PET/CT and ^68^Ga-DOTATATE PET/CT which helped to make a conclusive diagnosis that the lesion in the gastric antrum was an ectopic insulinoma.

**Interventions:**

The patient was cured with minimally invasive laparoscopic resection of the tumor.

**Outcomes:**

The symptoms were relieved and the blood glucose level remained normal after surgery.

**Conclusions:**

This case shows that ^68^Gallium-exendin-4 PET/CT is useful for precise localization and thereby successful treatment of insulinoma, especially for occult insulinomas and those derived from an ectopic pancreas.

## 1 Introduction

Insulinomas are the most frequent functioning pancreatic neuroendocrine tumors (NETs) with an estimated annual incidence of 0.4 cases per 100000 population.^[1]^ Ectopic insulinomas are extremely rare, accounting for about 1% of all insulinomas.^[2]^ Biochemical diagnosis of insulinomas is well established when hypoglycemia, supported by the presence of Whipple's triad, is associated with endogenous hyperinsulinism. However, the localization diagnosis is the key to successful surgical treatment. If conventional imaging examinations failed to find a lesion in the pancreas, searching for the tumor was almost an impossible task for clinicians in the past. Recently, the advent of radionuclide-based molecular imaging probes for β-cells opens the possibility of accurate localization of both orthotopic and ectopic insulinomas. Here we described a young patient with hyperinsulinemic hypoglycemia whose conventional imaging examinations revealed multiple suspected lesions both inside and outside of the pancreas. Finally, a conclusive diagnosis of ectopic insulinoma in the gastric antrum was made with the help of ^68^Ga-Exendin-4 PET/CT. The patient was cured with minimally invasive laparoscopic resection of the tumor.

## 2 Case presentation

A 23-year-old man with recurrent palpitation and sweating after activities for more than 1 year was admitted to our hospital in September 2020. His symptoms could be improved after eating. 1 year ago, he was sent to the emergency department of a local hospital because of confusion and sweating, with his blood glucose determined as 2.8mmol/L. The upper abdominal MRI scan did not reveal abnormalities, and the Insulin autoantibody, Islet cell antibody, and glutamate decarboxylase antibody were negative. Oral glucose tolerance test (OGTT) showed delayed insulin secretion. He was diagnosed with prediabetes and discharged. Since then, he increased food intake and avoided exercises, and the symptoms did not recur. However, he gained 25 kg in 1 year. After admission, a fasting test was performed and was stopped after 4 hour because of hypoglycemic attack. Serum glucose level was 1.7 mmol/L, insulin 53.3 mU/L, C-peptide 7.88 ng/ml, and the insulin release index 1.74. During his hospitalization, the nadir of blood glucose levels was 1.6 mmol/L, insulin 72.9 mU/L, and C-peptide 8.79 ng/ml, and the insulin release index 2.53. The simultaneous cortisol level was 31.3 ug/dl (5–25), Adrenocorticotropic hormone 45.1 pg/ml (0–46), growth hormone 7.940 ng/ml (0.06–5.0). A 5 hour-OGTT (Table 1) showed a lowest blood glucose level of 1.79 mmol/L at 5 hour, an insulin level of 84.3 mU/L, and a C-peptide level of 7.92ng/ml. Endogenous hyperinsulinemic hypoglycemia was confirmed, and an insulinoma was highly suspected. In terms of multiple endocrine neoplasia type 1 screening, pituitary hormones, parathyroid hormone level, and calcium level were not elevated. Pituitary MRI did not reveal any abnormality. Therefore, there was no evidence for a diagnosis of multiple endocrine neoplasia type 1.

**Table 1:** Preoperative and postoperative 5 h-OGTT.

		fasting	0.5 h	1 h	2 h	3 h	4 h	5 h
glucose (mmol/L)	preoperative postoperative	3.29 5.74	9.33 7.79	7.32 6.19	6.09 7.55	3.98 6.89	2.07 6.69	1.79 7.31
insulin (mU/L)	preoperative	17.4	192	125	112	81.9	46.9	84.3
	postoperative	14.3	53	20.6	75.1	38.7	36.9	49.7
C-peptide (ng/ml)	preoperative	2.82	11.8	10.5	10.4	9.52	6.01	7.92
	postoperative	3.14	7.27	5.07	8.38	6.12	5.12	6.14

**Figure 1:**
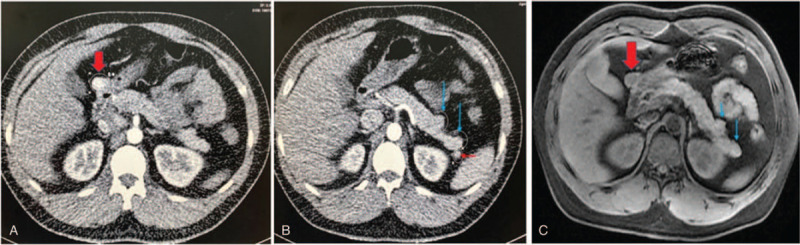
Pancreatic VPCT (A, B) and MRI (C) of the patient. (A)The thick arrow indicated the nodule on the right side of the gastric antrum (maximum cross-section of approximately 21 mm × 16 mm). (B)The thin arrows showed 2 small nodules in the tail of the pancreas (13 mm and 7 mm in diameter, respectively), and the short arrow showed a nodule in the hilum of the spleen. (C) The thick arrow indicated the nodule on the right side of the gastric antrum, and the thin arrows showed 2 small nodules in the tail of the pancreas.

**Figure 2:**
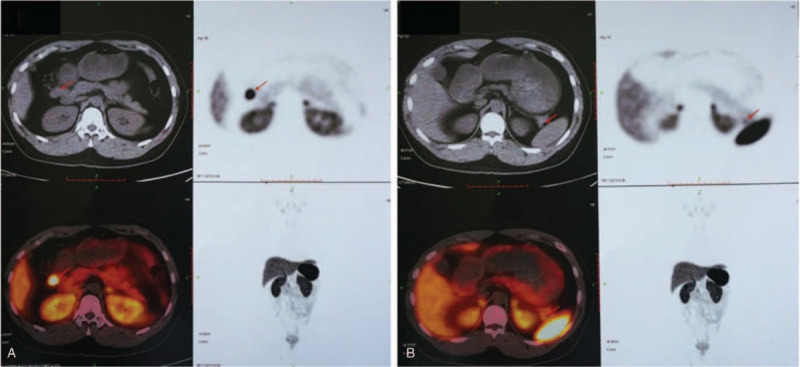
^68^Ga-DOTATATE PET/CT showed increased uptake of tracer in the nodule on the right side of the gastric antrum, no abnormal concentration of tracer was seen in the pancreas (A). The nodule in the tail of the pancreas showed no abnormal concentration of tracer (B).

Pancreatic volume perfusion CT (VPCT) revealed a small nodule on the right side of the gastric antrum (maximum cross-section of approximately 21 mm × 16 mm) (Fig. 1A) and 2 small nodules in the tail of the pancreas (13 mm and 7 mm in diameter, respectively) (Fig. 1B). All 3 nodules showed early enhancement in the arterial phase, islet cell tumors were suspected. A small nodule could be seen in the hilum of the spleen, the degree of enhancement was the same as that of the normal spleen, considered to be the accessory spleen. MRI (Fig. 1C) also showed an abnormal signal shadow on the right side of gastric antrum, and 2 nodules in the tail of pancreas. VPCT and MRI revealed multiple suspected lesions both inside and outside the pancreas. To determine the cause of hyperglycemia, we next performed ^68^Ga-DOTATATE PET/CT.

^68^Ga-DOTATATE PET/CT (Fig. 2) showed that a soft tissue nodule on the right side of the gastric antrum, about 2.0cm∗1.5 cm in size, had an abnormal concentrated tracer (SUV: 48.9). A small nodule in the hilum of spleen, about 0.9∗0.7 cm in size, with no abnormal concentration of tracer, was considered as an accessory spleen. No abnormal concentration of tracer was detected in the pancreas. The nodule on the right side of the gastric antrum was somatostatin receptor avid, suggesting a neuroendocrine tumor. We further performed ^68^Ga-Exendin-4 PET/CT to verify if it was an ectopic insulinoma.

**Figure 3:**
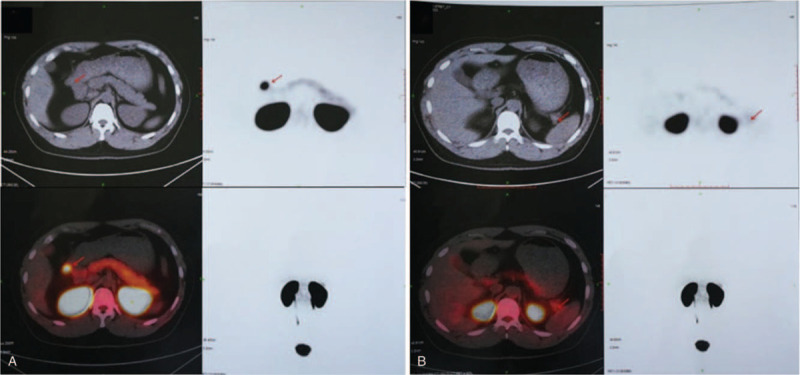
^68^Ga-Exendin-4 PET/CT showed intense uptake in the nodule on the right side of the gastric antrum, suggesting an insulinoma, no abnormal concentration of tracer was seen in the pancreas (A). The nodule in the tail of the pancreas showed no abnormal concentration of tracer (B).

^68^Ga-Exendin-4 PET/CT (Fig. 3) showed the nodule on the right side of the gastric antrum was also Glucagon Like Peptide 1 Receptor avid, with a SUV of 35.1. No abnormal concentration of tracer was detected in the nodule in the hilum of spleen and the pancreas. ^68^Ga-Exendin-4 PET/CT verified that the nodule on the right side of the gastric antrum was an ectopic insulinoma. To evaluated the metabolic activity of the lesions, we performed ^18^F-FDG PET/CT.

**Figure 4:**
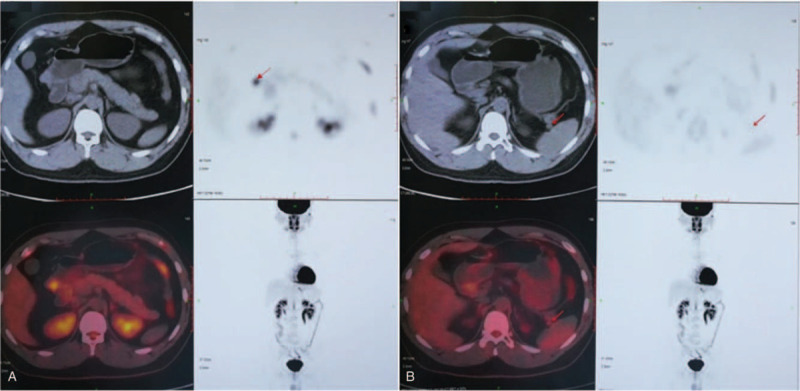
^18^F-FDG PET/CT showed abnormal concentration of tracer in the lesion on the right side of the gastric antrum (A). The nodule in the tail of the pancreas showed no abnormal concentration of tracer (B).

^18^F-FDG PET/CT (Fig. 4) showed the nodule on the right side of the gastric antrum with abnormal concentration of tracer, and the SUV was 5.4. It was considered a neoplastic lesion. Malignant lesion could not be excluded. The small nodule in the hilum of spleen had no abnormal concentration of tracer.

Ultrasound endoscopy showed an uneven hypoechoic mass on the outer side of the stomach wall, closely related to the gastric serosal layer. Elastography was blue-green, and the size was 1.5∗1.5 cm. Neuroendocrine tumor was suspected. Repeated ultrasound of the pancreas showed no obvious abnormal echo areas.

**Figure 5:**
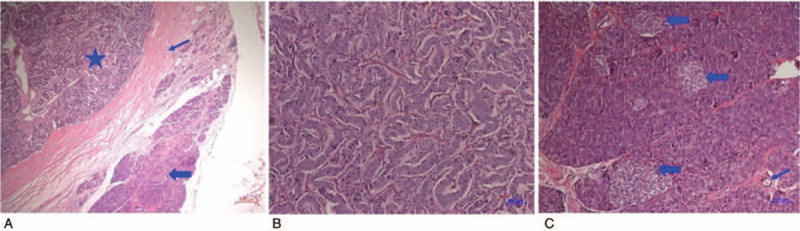
(A) The tumor is separated from the exocrine pancreas by an envelope. Star indicates the tumor, the thin arrow indicates the envelope, and the thick arrow indicates the exocrine pancreas. (B) The microscopy of tumor cells. (C) Islets (thick arrows) and duct (thin arrow) in the exocrine pancreas.

**Figure 6:**
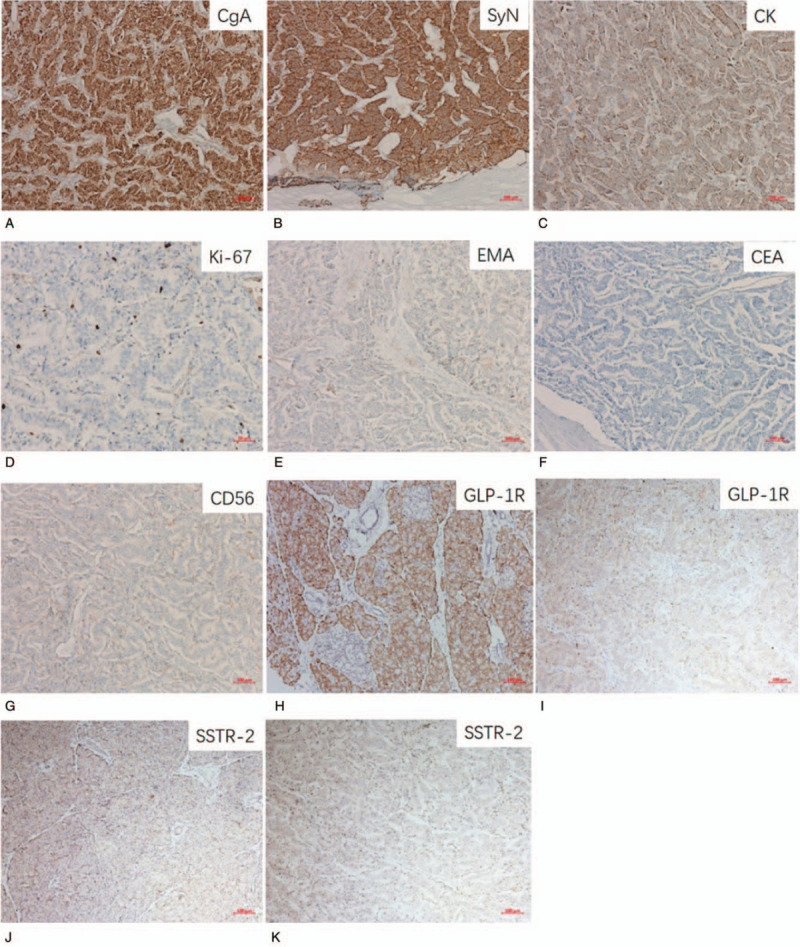
(A-G) Immunohistochemical staining of the tumor showed: chromogranin A (+), Synaptophysin (+), Cytokeratin (+), Ki-67 (index approximately 3%), and Carcinoembryonic Antigen, Epithelial Membrane Antigen, CD56 (−). The GLP-1R and SSTR-2 immunostaining showed that both the exocrine pancreas (H, J) and the tumor (I, K) expressed these receptors.

**Figure 7:**
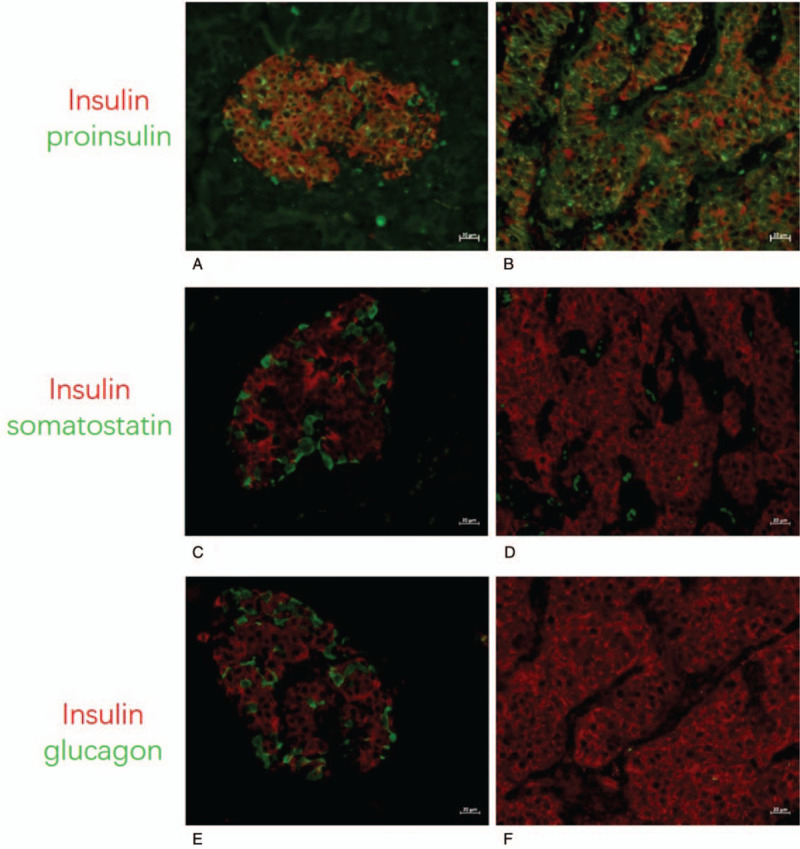
Immunofluorescence results showed that the islets in the exocrine pancreas (A, C, E) were positive for insulin, proinsulin, glucagon, and somatostatin. The tumor (B, D, F) only expressed proinsulin and insulin, confirming that it was an insulinoma.

The preoperative examinations strongly suggested an ectopic insulinoma located in the gastric antrum. The patient underwent laparoscopic wedge resection of gastric antrum mass under general anesthesia. Postoperative paraffin-embedded tissue pathology showed a (gastric antrum) neuroendocrine tumor (G2) with a distinct border surrounding exocrine pancreatic tissue (Fig. 5). The immunohistochemical staining of the tumor showed: chromogranin A (+), Synaptophysin (+), Cytokeratin (+), Ki-67 (index approximately 3%), and CD56, Epithelial Membrane Antigen, Carcinoembryonic Antigen (−) (Fig. 6A–G). The GLP-1R and SST-2 immunostaining showed that both the exocrine pancreas (Fig. 6H, J) and the tumor (Fig. 6I, K) expressed these receptors, in accordance with the positive results of ^68^Ga-Exendin-4 PET/CT and ^68^Ga-DOTATATE PET/CT. Immunofluorescence results (Fig. 7) showed that the islets in the exocrine pancreas were composed of β cells (insulin and proinsulin positive), α cells (glucagon positive), and δ-cell (somatostatin positive), While the tumor was only positive for insulin and proinsulin, confirming that it was an insulinoma. Repeated 5-hour OGTT showed no episodes of hypoglycemia 5 days after surgery (Table 1) the patient's glycemia remained normal 3 months after surgery.

## 3 Discussion

Insulinoma is a functioning pancreatic neuroendocrine neoplasm composed of insulin-producing and proinsulin-producing cells, with uncontrolled insulin secretion causing a hypoglycemia syndrome. It is the most common functioning pancreatic neuroendocrine neoplasms with an estimated annual incidence of 0.4 cases per 100000 population.^[1]^ Ectopic insulinoma is extremely rare, accounting for about 1% to 2% of all insulinomas. There are only a few cases reported in the literature. The diagnosis of this disease can be quite challenging because of the lack of effective localization diagnostic methods. With the development and maturation of radionuclide-based molecular imaging probes for β-cells, reports of ectopic insulinomas increased significantly in the last decade. We searched the English literature of the past 40 years in PubMed and found 13 patients who had this disease. Their data were listed in Table 2.

**Table 2:** Summary of patients with ectopic insulinoma reported in the literature and in this study.

Year	Authors	History	Glucose mmol/L	insulin mU/L	C-peptide	Gender/age	Location	Size	Grade	Metastasis	Ectopic pancreas	Insulin staining	Localization method	Procedure
1986	Miyazaki et al^[16]^	2.5y	1.7	174.5	NA	F/47	distal duodenum	2.1 × 1.8 × 1.3	NA	No	No	+	Duodenoscopy	Distal duodenum excised
2005	Hennings et al^[17]^	1y	1.6	70	2.8 nmol/l	F/74	adjacent to the duodenum (Treitz ligament)	3	NA	No	Yes	+	SSTR SPECT	enucleation
2009	Cardenas et al^[9]^	NA	2.1	53	4.4 ng/ml	M/46	intrasplenic heterotopic pancreas	7.6 × 4.4	NA	perineural and vascular invasion LN metastasis	Yes	+	MRI	splenectomy
2009	Christ et al^[18]^	6 m	2.1	38.6	1.7 nmol/L	M/64	Mesentery between duodenum and superior mesenteric artery	1.3	NA	NA	NA	+	^111^In-DOTA-exendin-4 SPECT in combination with CT scans	enucleation of the tumor
2011	Xian-Ling et al^[19]^	3y	2.07	16.3	1.62 nmol/L	M/21	Duodenohepatic ligament	1	NA	No	Yes	+	CT and DSA	wedge resection of the tumor
2013	La Rosa et al^[20]^	NA	2.4	5	1 ng/mL	F/75	duodenum	1.5	G1	No	No	+	CT and EGDS	enucleation
2015	Liu et al^[21]^	9m	2.4	35.4	2.5 ng/ml	F/79	retroperitoneum under the hepatoduodenal ligament	2.5	NA	No	Yes	+	CT	enucleation
2018	Li et al^[12]^	NA	1.5	>50	NA	F/53	pelvis	NA	G1	five lesions in the pelvis	No	+	68Ga-DOTA-NOC-PET/CT	removal of the five lesions
2018	Liu et al^[22]^	6y	3.4	193.3	9.6 ng/ml	F/31	proximal jejunum	3.5	G1	multiple liver metastasis	NA	+	CT and 68Ga-Exendin-4 PET/CT	everolimus plus longacting SSA octreotide
2019	Sun et al^[23]^	2y	1.37	6.69	1.99 ng/ml	F/37	gastrosplenic ligament	1.2	G2	No	NA	+	CT, MRI, 68Ga -Exendin-4-PET/CT	locally excised
2019	Sun et al^[23]^	3y	1.6	16.15	NA	F/51	adjacent to the duodenum	2.5	G1	No	NA	+	CT, somatostatin receptor scintigraphy and 99mTc-HYNIC-TOC SPECT/CT	locally excised
2020	Robin et al^[24]^	5Y	1.1	23.5	4.44 ng/ml	F/38	Proximal jejunum	1.9 × 1.8	G1	No	NA	NA	^68^Ga-DOTATATE PET/CT, ^68^Ga-Exendin-4 PET/CT, and CT	locally excised
2020	This case	1y	1.79	53.3	7.88 g/ml	M/23	Gastric antrum	2.1 × 1.6	G2	No	Yes	+	^68^Ga-DOTATATE PET/CT ^68^Ga-Exendin-4 PET/CT	locally excised

DSA = Digital Substraction Angiography, EGDS = esophagogastroduodenoscopies, F = female, LN = Lymph Node, M = male, NA = not applicable.

Typical Whipple triad and endogenous hyperinsulinemic hypoglycemia lead to a suspected diagnosis of insulinoma. However, the difficulty of diagnosis of insulinomas lies in the localization of the tumors which is also the key to the success of surgical treatment. Insulinomas are evenly distributed in the pancreas or show a slight predominance in the head and tail regions. Searching for the tumors is very challenging for clinicians. A relatively high number of insulinomas could not be found by intraoperative inspection and palpation by the surgeon. The sensitivity of contrast CT and MRI, 2 traditional noninvasive imaging examinations used to locate insulinoma, has been reported to range between 33% to 64% and 40% to 90%, respectively.^[3,4]^ The variability is dependent on tumor size, type of machine, protocols used, and the expertise of radiologists. In most of the cases (over 90%) the diameter of tumors is less than 2 cm, making it very difficult to accurately localize using MRI and CT prior to surgical excision. Moreover, multiple insulinomas, although rare (less than 10%) and often associated with MEN1, and ectopic insulinomas are even harder to accurately locate, and incomplete resection may cause symptom persistence. So like the patient in our study, quite a few patients suffered from a long history and repeated hospitalization because of the insensitivity of conventional localization examinations in the past. In the patient of our study, VPCT detected 2 nodules in the tail of the pancreas, a nodule in the gastric antrum, and a nodule in the hilum of the spleen. We first highly suspected that the 2 lesions in the tail of the pancreas where insulinomas located with a slight predominance were the cause of hypoglycemia. However, the nodule in the gastric antrum which was considered a NET by ultrasound endoscopy also showed early enhancement in the arterial phase in VPCT and had the same signal with normal pancreas in MRI, suggesting the possibility of ectopic insulinoma or a metastatic lesion, although insulinomas rarely spread to stomach. Besides, the nodule in the hilum of the spleen needed further examination. Therefore, multiple insulinomas, ectopic insulinoma, and metastatic insulinoma were suspected in this patient. No conclusive diagnosis could be made based on VPCT and MRI.

Glucagon Like Peptide 1 Receptor (GLP-1R) is overexpressed by up to 5 times in almost all benign insulinoma compared to normal human β-cells.^[5,6]^ Utilizing radionuclide-based exendin-4 (a stable synthetic GLP-1R agonist) imaging opened the possibility of highly specific imaging and accurate localization. ^68^Ga-labeled exendin-4 PET/CT, showed an excellent sensitivity of 95% to 98% and a distinct advantage over the traditional imaging modalities. In our patient, with the aid of ^68^Ga-Exendin-4 PET/CT, we could remove the suspect of the 2 nodules in the tail of the pancreas and the nodule in the hilum of the spleen and finally confirmed that the nodule in the gastric antrum with abnormally concentrated tracer was an ectopic insulinoma. 4 of the 13 cases of ectopic insulinomas we reviewed were diagnosed by ^68^Ga-Exendin-4 PET/CT, and the smallest tumor was 1.2 cm in diameter. This specific functional scan for insulinoma had high sensitivity for hidden small insulinoma, and helped to make a conclusive diagnosis when ectopic insulinoma was suspected.

In our patient, the tumor also took up high level of ^68^Ga-DOTATATE, a somatostatin analogue with highest affinity for somatostatin receptor 2. SSTs are overexpressed in neuroendocrine tumors, and SST PET has been suggested to be the preferred imaging modality for initial diagnosis of neuroendocrine tumors.^[7]^ Moreover, somatostatin imaging plays a role as a theranostic investigation to determine suitability for targeted radiotherapy with somatostatin analogs, which is known as peptide receptor radionuclide therapy. However, recent studies suggested that the expression of SSTs in benign insulinomas was less frequent and intense than GLP-1R,^[5]^ while most malignant insulinomas had high expression levels of SSTRs. Besides, the ^18^F-FDG PET/CT showed the SUV of the nodule in the gastric antrum was 5.4, and malignant lesion could not be excluded. Enhanced FDG uptake by high-grade tumors due to Warburg effect is a hallmark of in vivo tumor imaging with FDG PET/CT. The relationship of molecular markers with malignancy tendency in insulinomas was described as the “triple-flop” phenomenon, an increasing tendency toward malignancy during progression from GLP-1R avid (benign), to somatostatin receptor avid (malignant well-differentiated), to FDG avid (malignant poorly differentiated) insulinomas.^[8]^ Because the 3 imaging examinations of our patient were all positive, regular follow-up is needed in case of recurrent or metastatic disease.

With regard to ectopic insulinoma, no precise definition was provided in the literature, the case reports roughly included several categories. The most recognized definition is an insulinoma found in an ectopic pancreas. An ectopic pancreas is a rare entity defined as the presence of pancreatic tissue without any anatomic or vascular continuity with the pancreas. It may occur at a variety of sites in the gastrointestinal tract having a propensity to affect the stomach and small intestine. Ectopic insulinomas within an ectopic pancreas were clearly described in 5 patients in our literature review. The most common site was duodenum and adjacent tissues, such as duodenal ligament, spleen, Treitz ligament and proximal jejunum. The tumor localization in this study was gastric antrum, which was also adjacent to duodenum. Other location, such as spleen, was also reported.^[9]^

The second category is non-functioning gastrointestinal neuroendocrine tumors that gradually achieve the ability of insulin secretion and cause hypoglycemia. Although without clinical syndrome, about 40% of non-functioning NETs produce multiple hormones. The hormones most often expressed are somatostatin, pancreatic polypeptide, and glucagon. In a series of 61 nonfunctioning tumors,^[10,11]^ PP immunoreactivity were detected in 35% of cases, with a mean of 33% of all tumor cells; glucagons were found in 30% of cases and 30% of cells; somatostatin in 15% of cases and 20% of cells; serotonin in 20% of cases and 36% of cells; calcitonin in 20% of cases and 10% of cells; and insulin in 15% of cases and 2% of cells. No gastrin or vasoactive intestinal peptiepeptide immunoreactivity was detected. It was reported that 6 years after excision of a rectum G1 neuroendocrine tumor in a middle-aged woman, 5 pelvic lesions which might transit from rectum NET acquired the ability of insulin secretion and caused hypoglycemia.^[12]^ Both the rectum tumor and the 5 pelvic foci showed expression of insulin, while the former did not cause any symptom. How does non-functioning NETs acquire the ability of insulin secretion during the course of disease is unknown.

Some non-islet-cell tumors have been reported to secrete insulin and cause hyperinsulinemic hypoglycemia. Among them are bronchial carcinoid tumor, squamous-cell carcinoma of the cervix, neurofibrosarcoma, schwannoma, paraganglioma, small-cell carcinoma of the cervix, and gastrointestinal stromal tumor.^[13]^ Some studies clearly showed that non-islet-cell tumors were the origin of the hyperinsulinemia on the basis of the detection of proinsulin mRNA and insulin protein within the tumor cells. In this study, these tumors were not classified as an ectopic insulinoma.

The majority of insulinomas are benign, only about 10% are malignant.^[14]^ The only malignancy criterion defined by World Health Organization (WHO) for insulinoma is the presence of metastases,^[15]^ being the liver the most frequent affected organ.^[14]^ Of the 13 ectopic insulinomas we summarized, 2 patients with liver metastases and lymph nodes metastases respectively could be confirmed as malignant insulinoma. However, because of the scarcity of cases, it was not reliable to conclude the malignant tendency of ectopic insulinomas. Most insulinomas are less than 2 cm in diameter, but more than half of the ectopic insulinomas we have reviewed from the literature were greater than 2 cm (7/13) in diameter. This may be related to the long course of disease (mean history: 29.7 months), delayed diagnosis, and the existence of exocrine pancreas.

In summary, we reported a case of ectopic insulinoma in gastric antrum. Precise preoperative localization and functional examinations with ^68^Ga-Exendin-4 PET/CT is essential to the localization diagnosis and precision surgery of this disease. ^68^Ga-DOTATATE PET/CT and ^18^F-FDG PET/CT are also helpful in characterizing the tumor and assessing the prognosis. Further investigation needs to focus on the biological behavior of ectopic insulinomas.

## Author contributions

XZ, HJ, and FL collected, analyzed and interpreted the clinical data. HJ provided the pathology analysis. CF and JZ performed immunohistochemical and immunofluorescent staining of the tumor. ML reviewed the literature. XZ wrote the initial draft for this case. QH and ML critically revised the manuscript. All authors have read and approved the manuscript.

**Conceptualization:** Ming Liu.

**Data curation:** Xiaona Zhang, Hongwei Jia, Fengao Li, Chunyun Fang.

**Funding acquisition:** Xiaona Zhang.

**Investigation:** Hongwei Jia, Fengao Li, Qing He, Ming Liu.

**Methodology:** Chunyun Fang, Jinyang Zhen.

**Supervision:** Hongwei Jia, Qing He, Ming Liu.

**Writing – original draft:** Xiaona Zhang.

**Writing – review & editing:** Qing He, Ming Liu.
